# Hyperscanning EEG Paradigm Applied to Remote vs. Face-To-Face Learning in Managerial Contexts: Which Is Better?

**DOI:** 10.3390/brainsci13020356

**Published:** 2023-02-18

**Authors:** Michela Balconi, Laura Angioletti, Federico Cassioli

**Affiliations:** 1 International Research Center for Cognitive Applied Neuroscience (IrcCAN), Università Cattolica del Sacro Cuore, 20123 Milan, Italy; 2 Research Unit in Affective and Social Neuroscience, Department of Psychology, Università Cattolica del Sacro Cuore, 20123 Milan, Italy

**Keywords:** face-to-face learning, e-learning, hyperscanning, EEG, cognitive and affective process

## Abstract

We propose a hyperscanning research design, where electroencephalographic (EEG) data were collected on an instructor and teams of learners. We compared neurophysiological measures within the frequency domain (delta, theta, alpha, and beta EEG bands) in the two conditions: face-to-face and remote settings. Data collection was carried out using wearable EEG systems. Conversational analysis was previously applied to detect comparable EEG time blocks and semantic topics. The digitalization of training can be considered a challenge but also a chance for organizations. However, if not carefully addressed, it might constitute a criticality. Limited research explored how remote, as opposed to face-to-face, training affects cognitive, (such as memory and attention), affective, and social processes in workgroups. Data showed an alpha desynchronization and, conversely, a theta and beta synchronization for the face-to-face condition. Moreover, trainees showed different patterns for beta power depending on the setting condition, with significantly increased power spectral density (PSD) in the face-to-face condition. These results highlight the relevance of neurophysiological measures in testing the e-learning process, in relation to the emotional engagement, memory encoding, and attentional processing.

## 1. Introduction

The massive digitalization of the learning process happening in professional environments represents an innovation [1]. It is known that some assets remain unrecorded on an organization’s balance sheet even though they still present significant economic value [2]. In the long term, these resources guarantee the company’s competitiveness in complex environments. Hence, the aim of Learning and Development (L&D) is to sustain the company’s evolution by allowing the employees’ professional growth. Workgroups more and more learn in remote conditions [3] and are now often geographically apart [4].

Thus, both academics and executives naturally came up with a comparison between online and face-to-face learning [5,6].

Given the relevance of the research topic, in the current study, we decided to explore this distinction. How does the conversion of a training experience, from face-to-face to remote, affect the learning experience? Does the digitalization of social interaction have positive vs. negative consequences at individual and interpersonal levels? Current literature presents several plausible theoretical arguments that are now reported and discussed. Unfortunately, they tend to appear ambiguous, if not contradictory at times [7]. Thus, we consider available evidence not conclusive.

Overall, three major distinctions between face-to-face and remote conditions were highlighted: the discrepancy in the role of technology, a difference in the needed required tasks for the participant, and a divergence in the engagement levels. Regarding the first two factors, we might consider them technical-oriented. Remote learning is inherently rooted in the employment of software that permits the development of a shared space (if not a place) for learning. Further, individuals in online environments have to handle technical specifics (audio-video settings, personal space, and surroundings) and more stimuli from devices compared to face-to-face settings (e.g., notifications). Moreover, a motivational difference for the learner was highlighted based on the setting condition. Distance training seems to prompt lower satisfaction and work engagement, together with a tendency to be non-responsive, with decreased empathy [8]. Regarding the miscellaneous communication difficulties that inhibit the learner–instructor interaction, the nonverbal components (such as facial expression, body appearance, and movements) tend to be limited in remote condition and even minor signal delays force an individual’s brain to restore the desired synchrony [9]. Additionally, augmented use of cognitive resources for registering and perceiving the communication of others was found [10]. This phenomenon could be associated with the quantity of close-up gaze, the increased cognitive load and self-evaluation from looking at oneself in the video, and mobility constraints [11]. Lastly, it was found that four out of five American students attending online classes experienced isolation, depression, and anxiety, and found it harder to focus and stay in the moment [12]. It should be noted that these data were gathered during COVID-19, and this could be a possible explanation, as noted by the authors.

Unfortunately, other available data also depicted a different scenario, this time in favor of e-learning. For instance, a previous work on telecommunication displayed that remote condition is related to decreased cognitive demand [13] and that some individuals might find social face-to-face interactions more stressful because of personal traits [14].

As we showed, this comparison appears controversial. Distance learning can be portrayed as a double-edged sword [15]. An in-depth investigation might represent an opportunity to assess it. In this regard, we advocate for the attention of three methodological cues.

Firstly, learning is an interindividual process. Acknowledging it means embracing its complexity as social interaction and its investigation should simultaneously consider all involved agents [16]. Secondly, the limited use of multidisciplinary approaches to study learning is a well-known weakness in the literature [17]. A delicate balance between the use of quantitative metrics without undermining ecological validity is a priority. Thirdly, self-report and qualitative measures alone might result feeble in investigating covert processes, such as attentional and affective ones. Additionally, via these methods, biases could incur (e.g., a-priori or ideological viewpoint on technology). For the issues here discussed, we advocate for the consideration of cognitive neuroscience which represents a valuable perspective to assess education and learning at a neurophysiological level [18].

Historically, neuroscience considered heterogeneous factors affecting the learning process, such as diet, physical exercise, rest, and level of relaxation [19]. Since, in this work, we consider learning as an interindividual process, social neuroscience should be naturally called into question. In particular, we found hyperscanning as an innovative methodological approach that could come in handy for the assessment of remote learning. The hyperscanning paradigm allows the contemporaneous data recording of more individuals involved in a shared task, activity, or simulation of a real condition [20]. The synchronization of the electrophysiological activity within a couple or a group represents highly valuable data because it allows the consideration of an individual in a social environment or a dynamic.

The analysis of the neurophysiological correlates of social interactions in classrooms was conducted before [21]. Generally, two measures are derived from an EEG hyperscanning design, intra-brain, and inter-brain connectivity. Intra-brain connectivity refers to the neural synchronization between different cortexes within a participant and can be considered as a marker for a subject’s functional specialization [22]. The other indicator is inter-brain connectivity [23], which refers to the functional connectivity between individuals’ brain regions responsible for interpersonal coupling during social interactions [24,25]. Interestingly, these metrics are both good predictors of collective performance [26]. In electrophysiological studies, EEG spectral boundaries are considered (i.e, delta, theta, alpha, and beta bands) and interpreted according to their functional meaning that is related to specific cognitive and emotional functions or processes [27]. Such patterns are shown to be correlated to cognitive and affective processes and are commonly employed in neuroscientific research designs. Low-frequency bands (delta and theta) were shown being associated with emotional and memory-related processes [28,29], during social feedback [30], and emotional face comprehension [31]. Furthermore, alpha desynchronization and beta synchronization are frequently linked to cognitive engagement and processes of selective attention toward target stimuli (see [32]).

For the reasons previously described, we intended to develop a research design, gathering data for the investigation of the learning experience of trainees and trainers, eliciting possible discrepancies between the remote and face-to-face conditions. EEG allows the consideration of quantitative metrics that refer to cognitive load, cognitive arousal and mental stimulation, and affective states. The present neurocognitive paradigm aims at furnishing a comparison of face-to-face and remote settings for learners and a trainer during two equal training sessions. Preserving ecological validity was a primary condition and, to ensure it, we chose to employ EEG wearable devices. A possible methodological issue we encountered was the homogeneity between the two conditions (i.e., face-to-face vs. remote) with a sufficient degree of reliability. To extract comparable phases to match the neurophysiological data, previous studies qualitatively detected recurring verbal patterns in inter-agent conversational interactions [33]. In this sense, blending EEG and conversational data might represent a valuable methodological approach for the assessment of training sessions.

To achieve the objectives, EEG data were collected in three groups of trainees and a trainer, employing a hyperscanning paradigm in two continuous sessions, one provided remotely and the other in person. Moreover, to map the discourse and reoccurring topics in the two sessions, a qualitative content analysis was adopted. We then compared the neurophysiological data from the two sessions, taking into account the frequency powers of the delta, theta, beta, and alpha waves, from participants’ specific brain areas.

Given the research’s aims and the employed methods, we proposed the following hypotheses:

First, regarding theta and delta band power, it was hypothesized that the face-to-face condition exhibits higher power activation; that is, the higher presence of these EEG frequency bands previously associated to emotional engagement, compared to the remote one. In the first situation, when nonverbal language is also used by the participants to communicate, we expect stronger emotional involvement, as evidenced by a theta synchronization [28].

Instead, considering beta and alpha bands as markers of attention or cognition, we expect stronger synchronization in all training groups under those situations that require a higher attentional cost related to the specific phase of the learning process. It might be possible that, when trainees were attending the remote session they could be less responsive or less cognitively engaged and more susceptible to being distracted by their surroundings.

## 2. Materials and Methods

### 2.1. Sample

After giving their written informed consent, a total of eight participants [mean (M) age = 42.6, standard deviation (SD) age = 6.12] took part in the study. Professional trainers and trainees (*n* = 7 individuals) were included in the sample as two separate groups. The following inclusion criteria were taken into account for the trainer and trainees, respectively: being a senior trainer (with more than five years of expertise managing training and educational settings) and, for trainees, having experience of more than 5 years in human resources (HR) management.

Criteria of exclusion encompassed: (i) having a history of neurological or psychiatric conditions, (ii) utilizing psychoactive medications that affect the central nervous system concurrently with therapy, (iii) displaying clinically significant discomfort, or having experienced burnout. The research activity was conducted following the principles of the Helsinki Declaration (1964) and was approved by the local Ethical Committee Institution of the Department of Psychology, Catholic University of the Sacred Heart, Milan, Italy.

### 2.2. Procedure

**Figure 1:**
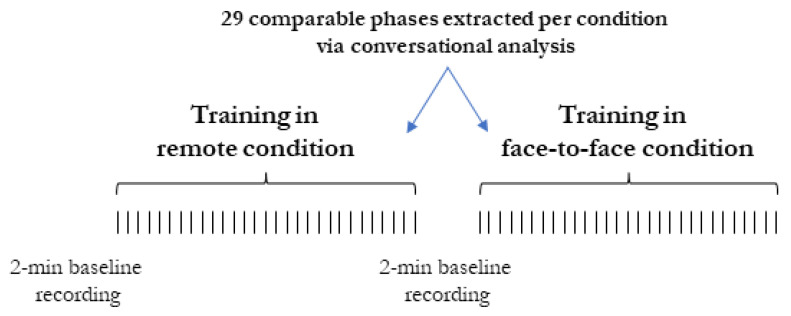
The procedural steps of the experiment. Comparable phases (*n* = 29 per condition) were extracted via conversational analysis. A 2 min baseline was recorded before each condition.

After agreeing to participate, each participant attended two training sessions offered by a counseling agency, one given remotely using Zoom Video Communications and the other in person. Both sessions were video-recorded and lasted 3 h: during these sessions, an HR trainer presented ways for delivering learning content in corporate contexts. Participants remotely attended the training session from their own laptops under the remote condition. A researcher made sure that each participant could concentrate on the instruction in a calm, low-light environment before the remote session began. Participants in the face-to-face condition were introduced in a classroom where training sessions are commonly conducted. Participants in both conditions were required to wear the EEG equipment so that their training related brain activity could be collected concurrently. All participants received wearable EEGs from the study team, which they paired with their cellphones using Bluetooth. All participants received training before of the trial and could independently wear the EEG system and record the session (start and stop options). A 120 s baseline was recorded previous to each condition. After each single session, the recorded data were saved and sent to the researcher. The output was then permanently deleted from the participant’s smartphone. The introduction and conclusion stages, as well as any requests about the usage of the device in both conditions, were overseen by a member of the research team who was continually on hand. The same experimental procedure was adopted in a previous study [34] and it is reported in Figure 1.

### 2.3. EEG Signal Acquisition

**Figure 2:**
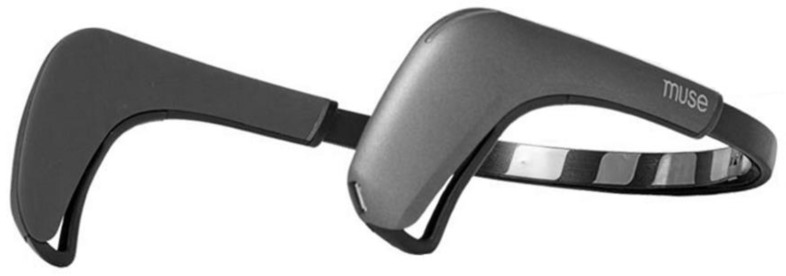
The wearable EEG systems employed in the study: Muse™ Headband version 2 (InteraXon Inc., Toronto, ON, Canada).

EEG data were acquired using multiple Muse™ Headbands version 2 (InteraXon Inc., Toronto, ON, Canada). These wearable recording systems (Figure 2) are composed of 4 gold-plated cup bipolar dry electrodes to non-invasively detect EEG signals.

The electrodes are positioned according to the international EEG placement system: three are used as a reference, and the other four are positioned in the frontal (AF7 and AF8) and temporoparietal (TP9 and TP10) regions. The system is also equipped with an accelerometer, a gyroscope, and pulse oximetry, and is connected to the participant’s smartphone, using the mobile application Mind Monitor [35]. Data were sampled at a constant of 256 Hz and a 50 Hz notch frequency filter is applied. The software automatically processes raw data and applies fast Fourier transform to obtain brain waves computing the logarithm of power spectral density (PSD) from each of the four channels (as processed by Mind Monitor, all EEG PSD values tend to lie within the −1: +1 range). Considered frequency bands were delta (1–4 Hz), theta (4–8 Hz), alpha (7.5–13 Hz), and beta (13–30 Hz).

### 2.4. Data Analysis

Data analysis included three phases: conversational semantic mapping; EEG frequency band analysis and statistical modeling, between participants (trainer and trainees) based on their EEG activity during the different phases.

#### 2.4.1. Conversational Semantic Mapping

The conversational semantic mapping of the stages of the process in which the participants were conversing was realized by exploiting the videotape data from the two sessions. Two impartial judges, who had transcribed the audio of the videotape, carried out the mapping. The transcripts were analyzed using a qualitative content analysis methodology [36]. Transcripts of the verbatim were also examined. Independent researchers checked the accuracy of dialogue transcription. In order to ensure that the transcripts accurately reflected the discourse, researchers repeatedly evaluated the verbatim and compared their findings with those of the other judge. The research tried to find similar themes in the two conditions’ material. After reading interview transcripts several times, a thematic analysis of their content was carried out, starting with a first coding process that involved identifying the recurring topics.

This analysis permitted to highlight how the verbatim material pertained to particular stages (*n* = 29) of the training procedure. The group 1, group 2, group 3, trainer, and feedback clusters were identified. According to the training objectives, the first three clusters (group 1 phase, group 2 phase, and group 3 phase) corresponded to the three-team stages presenting their project proposals. The fourth cluster (trainer) concerned the trainer, who was individually discussing and imparting methods and tools for a remote learning strategy. The fifth cluster (feedback) contained all of the instances where participants provided general comments on the training session.

The most important parts of the training process, present in both two training conditions (face-to-face and remote) were used to create a time-block for the analysis on the recorded EEG trace (see also [33]). The sample is constituted by the number of EEG samplings made per subject in the different experimental conditions.

#### 2.4.2. EEG Frequency Band Analysis

EEG recording was divided into time blocks according to the different salient phases of the training process for each cluster of phases. We then extracted the data of the corresponding electrophysiological activity per phase. Each of the five clusters contained the EEG signal corresponding to the different salient phases of the training process, grouped according to the following specific topic: group 1/2/3, trainer, and feedback. The average length of each topic was twenty minutes and was homogeneous among the topics.

For the statistical analysis, four ANOVA models were run, one per frequency band (delta, theta, alpha, and beta) dependent variable. The following independent variables were modeled: condition (2: face-to-face, remote) electrodes (4: AF7, AF8, TP9, and TP10) as within factor, and topic (group 1, group 2, group 3, trainer; feedback) as a between factor. Post hoc analysis (contrast analysis for ANOVA, with Bonferroni corrections for multiple comparisons) was successively applied to reduce type I error. The sphericity was assessed via Mauchly’s test and, when violated, the Greenhouse–Geisser correction was applied. The size of statistically significant effects was estimated via partial eta squared (η2) indices. Significant results for theta, alpha, and beta are reported and interpreted. No significant results were detected for the delta wave.

## 3. Results

### 3.1. Theta Band

**Figure 3:**
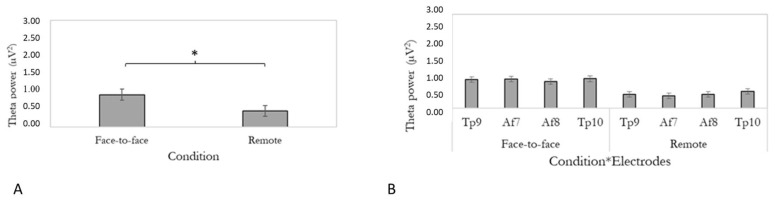
(**A**). The bar graph shows differences in theta power mean values for condition. Error bars represent ± 1 standard error (SE). Stars (*) mark statistical significance. (**B**)**.** Bar graph shows differences in theta power for the not-significant interaction condition*electrodes.

**Figure 4:**
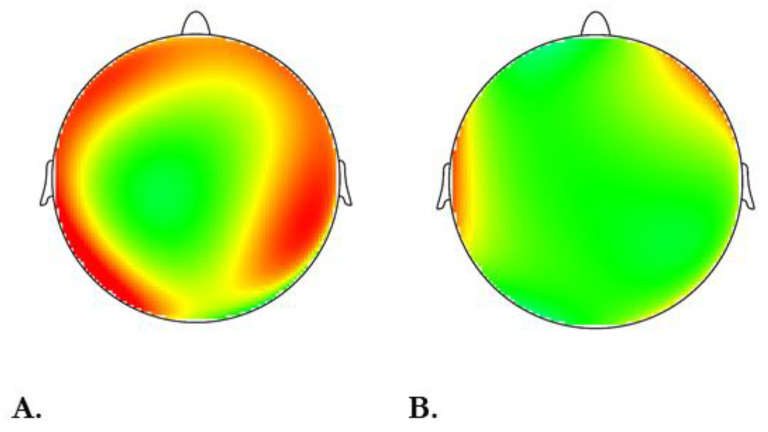
Theta power EEG display for face-to-face condition (left head, (**A**)) compared to remote condition (right head, (**B**)). For all EEG head displays, red represented an increase in power for the considered frequency band.

The effect of condition was found to be significant (F [1,24] = 14.43, *p* ≤ 0.05, η2 = 0.38). Specifically, we observed increased theta power in the face-to-face condition, compared to the remote setting. Data are reported in Figure 3A. We also reported the interaction condition*electrode. Right temporoparietal theta power in face-to-face conditions is descriptively higher than the anterior–frontal PSDs. Data are reported in Figure 3B. Theta power for the two conditions is also represented in two head displays in Figure 4A,B.

### 3.2. Beta Band

**Figure 5:**
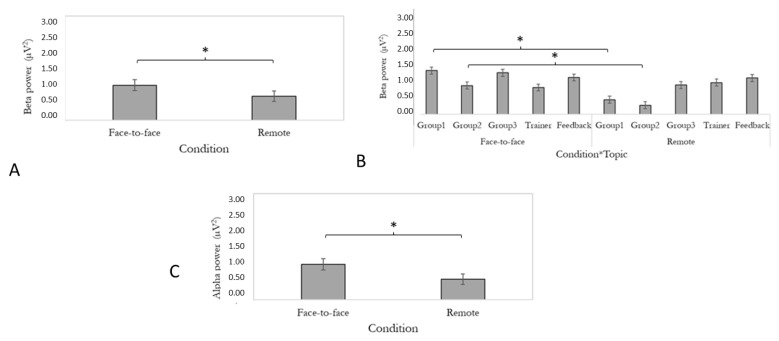
(**A**) Bar graph shows differences in beta power mean values for condition. Bars represent ± 1SE. Stars (*) mark statistical significance. (**B**) Bar graph shows differences in beta power mean values for the significant interaction condition*topic. Error bars represent ± 1SE. Stars (*) mark statistical significance. (**C**) Bar graph shows differences in alpha power mean values for condition. Error bars represent ± 1SE. Stars (*) mark statistical significance.

The effect of condition was found to be significant (F [1,24] = 14.43, *p* ≤ 0.05, and η2 = 0.38). Higher beta power was found in the face-to-face condition compared to the remote one. Data are reported in Figure 5A. Moreover, the interaction condition*topic was found to be significant (F [4,24] = 5.546, *p* ≤ 0.05, and η2 = 0.48). Post hoc analysis highlighted that in two group phases out of three there was a significant difference between face-to-face and remote conditions. Specifically, group 1 (*p* ≤ 0.05) and group 2 (*p* ≤ 0.05) showed increased beta power in the face-to-face condition compared to the remote one. Data are reported in Figure 5B.

### 3.3. Alpha Band

The effect of condition was found to be significant (F [1,24] = 9.38, *p* ≤ 0.05, and η2 = 0.28). We observed increased alpha in the face-to-face condition compared to the remote setting. Data are reported in Figure 5C.

## 4. Discussion

This research brings to the fore the advantages of applied neuroscience for the investigation of organizational training experience, where the investigation of the impact of the setting, considered in remote or face-to-face conditions was the main focus. During two training sessions, electrophysiological indices were collected to explore the cognitive and emotional states of a trainer and trainees. The analysis of the data encompassed three stages: discourse analysis, EEG data extraction within the frequency domain starting from the qualitative evaluation, and statistical modeling. The identification of compatible phases between the two considered conditions was made possible by the qualitative content analysis. As performed in previous studies [33], time blocks were created for the EEG recording based on the themes that emerged from the qualitative analysis. We then retrieved the corresponding electro-physiological activity for each participant based on these temporal segments. For the following frequency bands: theta, beta, and alpha PSD noteworthy evidence was observed.

As expected, the factor condition was found to be significant. In comparison to the remote situation, there was increased theta power activity in the face-to-face condition. Theta rhythm’s involvement in the control of emotion was solidly emphasized in the literature (e.g., [37,38,39]). Theta pattern is generally considered a global processing mode that spans large cortical regions, mostly for regulating spatially distributed neural assemblies (e.g., [40]). Consistent data suggest that during emotional arousal, neurons in the amygdala produce theta activity (e.g., [41]). Its role in interindividual communication, social feedback [30], and emotional processing is widely known [28,29]. Data showed that in the occurrence of immersive experience, theta tends to increase [42]. It is also associated with stress relief, memory recollection [43], and improved creativity and learning [44]. From the gathered EEG evidence, we state that face-to-face settings could elicite a better general learning experience, supporting already existing data previously introduced. This line of studies supports the idea of remote conditions weakening empathy levels (e.g., [8]) and work engagement [10], and ultimately affecting social interactions at an emotional level. Some explanatory factors could be related to interindividual issues, such as reduced facial expression patterns, body appearance, and miscellaneous body movements. Interestingly, even though at a descriptive level, temporoparietal loci (TP9, TP10), in particular the right one, presented higher powers. Converging neuroimaging evidence show that the right temporoparietal junction (rTPJ) is connected to social processing [45,46] and slow waves (i.e., theta) support TPJ functionality [47]. In particular, its activation seems to be linked to the processing of social cues required for empathy [48]. Although further investigation should address this evidence.

Regarding beta activity, we detected higher PSD when participants were experiencing the training in the face-to-face than in the remote condition. Significant differences were also observed in the two group phases out of three (groups 1 and 2), with increased beta power in face-to-face conditions. Generally, beta is considered a proxy for attentive accuracy, and the sensorial detection of internal and external stimuli [49]. The contribution of the cortico-thalamic supported by the projections from the lateral geniculate nucleus (LGN) and the primary visual cortex (V1) is crucial for attentional perception and higher visual processing. Additionally, the beta pattern tends to increase during awareness, concentration, and immersive tasks [42]. In this sense, the setting condition seems to play an important role in the modulation of attention. Interindividual differences within the groups were shown to have a certain weight on the learning experience, supported by the detected differences in the PSD. Instead, the trainer’s activity and the feedback exchange appeared unaffected by the condition factor.

Lastly, concerning alpha activity, we found a wave desynchronization in the face-to-face condition. The interpretation of alpha starts from Berger’s studies [50] as it was considered a reverse measure of activation, thus exhibiting a negative correlation with cognitive performance. Recent evidence suggests a reconceptualization of alpha, which is now mainly considered for its inhibition function, towards task-irrelevant or conflicting processes, and a mechanism for increasing the cortex signal-to-noise ratio [51,52]. Alpha oscillations decrease while experiencing concentration and immersion states [42]. Therefore, from the observations we gathered, it could be argued that the face-to-face condition allowed a better experience, cognitively, for participants, possibly experiencing an immersive learning process. From a learning efficacy perspective, alpha desynchronization was previously linked to memory encoding; thus, the face-to-face condition potentially allowed the participants to reach increased learning outcomes. This evidence is in line with literature suggesting a needed higher cognitive load for remote conditions, due to increased looking at one’s reflection and evaluating oneself, as well as physical limitations [10,11].

## 5. Conclusions

To sum up, this research, combining a conversational approach with EEG data, correctly addressed the set objectives and hypothesis. Cognitive and affective processes, such as selective attention, general cognitive arousal, memory, and social cues encoding, were considered in their general effect, revealing specific modulations for the condition factor.

The present work could present some strengths and weaknesses. We believe that the presented study adds value to the literature for three main reasons: First, the wearable EEGs used to evaluate the impact of the condition factor (remote vs. face-to-face) on the training experience is a novelty. Secondly, the combined approach used in this study (exploiting both quantitative and qualitative data) represents a strength point. We were able to successfully compare the two considered conditions, via an in-depth qualitative analysis of the conversation content, while employing hard metrics, such as electrophysiological correlates (i.e., EEG PSD per band) for covert processes. We believe that it represents a robust solution for the assessment of complex social interactions. Lastly, the research involved a real setting, trainer, and trainees in order to preserve ecological validity. Instead, the study’s limitations we identified are related to internal validity and external validity. Regarding the latter, the research generalizability might represent a weakness due to the inherent complexity of social interactions. Internal validity is also a dimension that should be further assessed. In fact, confounding variables are possibly present (e.g., learning content, individual variables, order effect of the condition). For these reasons, such preliminary findings should be replicated and further researched with appropriate samples via the computation of power analysis to establish the needed sample size. Lastly, the recorded signal could be analyzed through more sophisticated processing [53], while considering other sociodemographic factors and self-report variables (e.g., motivation and psychological traits), as well as a training approach. Additional contributing independent variables should be taken into account and adjusted for in order to increase statistical power.

Practical consequences can be considered as well. This evidence demonstrated that the environment may have an impact on how a training experience is designed.

When deciding on the specifics of the training, consideration for the targeted demographic of trainees should be given. Moreover, the used commercial EEG equipment proved to be effective for practitioners as well. Even with a limited technical background in evidence-based practices, these tools represent an affordable solution.

## Data Availability

The dataset is available from the corresponding author on reasonable request.
